# Comparison of prophylactic ipsilateral and bilateral central lymph node dissection in papillary thyroid carcinoma: a meta-analysis^☆^

**DOI:** 10.1016/j.bjorl.2023.101318

**Published:** 2023-09-04

**Authors:** Yujie Li, Lingling Lao

**Affiliations:** a Ningbo No.2 Hospital, Department of General Surgery, Ningbo, China; b Yuyao People’s Hospital, Department of General Surgery, Zhejiang Province, China

**Keywords:** Papillary thyroid carcinoma, Ipsilateral CND, Bilateral CND, Meta-analysis

## Abstract

**Objective:**

The scope of surgical resection for paratracheal (level VI) lymph nodes in patients with Papillary Thyroid Carcinoma (PTC) remains debatable. The aim of our study was to evaluate the effectiveness and safety of prophylactic ipsilateral Central Neck lymph node Dissection (CND) versus bilateral CND at the time of total thyroidectomy for Clinically Node-negative (cN0) unilateral PTC.

**Methods:**

A systematic retrieval of electronic databases, including Pubmed, Web of Science, and the China Journal Net, was conducted from January 1990 to September 2021. Outcome data of interest included transient hypoparathyroidism, permanent hypoparathyroidism, transient Recurrent Laryngeal Nerve (RLN) injury, permanent RLN injury and local recurrence. We constructed the summary Odds Ratios (ORs) and 95% Confidence Intervals (CIs) for every study with either fixed or random effect models.

**Results:**

A full total of 1792 patients from 6 studies were enrolled. Our meta-analysis showed that transient hypoparathyroidism was significantly more frequent in bilateral CND group (OR = 0.58; 95% CI 0.44–0.76). The prevalence of permanent hypoparathyroidism was significantly higher in bilateral CND group patients compared to those in ipsilateral CND group (OR = 0.26; 95% CI 0.15–0.45). On the other hand, our meta-analysis indicated that there were no significant differences in the incidence of transient RLN injury, permanent RLN injury and local recurrence.

**Conclusions:**

Compared with bilateral CND, the rate of temporary and permanent hypoparathyroidism in ipsilateral CND is lower, but the local recurrence is similar. It may be presumptuous to suggest that ipsilateral CND is an adequate treatment for cN0 unilateral PTC.

## Highlights

• The permanent hypoparathyroidism was remarkably higher in patients in the bilateral CND group compared to the ipsilateral CND group.
• Ipsilateral CND may be comparable to bilateral CND in terms of local recurrence and transient/permanent RLN injury.
• Ipsilateral CND may be an appropriate treatment forcN0 unilateral PTC.

## Introduction

Papillary Thyroid Carcinoma (PTC) is the most usual type of thyroid carcinoma, representing 80%‒90% of all thyroid cancer cases.^1^ The central cervical compartment is regarded as the main echelon of the PTC lymph node metastases. The rate of occult lymph node metastasis in patients experiencing prophylactic Central Neck Dissection (CND) is from 20% to 90%.^2^, ^3^, ^4^

Prophylactic CND is described as the resection of level VI lymph nodes, including the pre-tracheal, pre-laryngeal, and paratracheal lymph nodes on both sides of the trachea. Nevertheless, compared with total thyroidectomy alone, prophylactic CND does carry increased risks, which contains an increased incidence of hypoparathyroidism as well as Recurrent Laryngeal Nerve (RLN) injury.^5^

Ipsilateral dissection only removes the central lymph nodes on one side of the initial tumor, thereby reducing the likelihood of damage to the contralateral parathyroid and RLN. Whether ipsilateral CND can be regarded as an effective alternative remains to be proven. In this study, we meant to compare the results of ipsilateral CND and bilateral CND in patients with clinical unilateral Clinically Node-Negative (cN0) PTC.

## Methods

### Search strategy

Using the Preferred Reporting Items for Systematic Reviews and Meta-Analyses (PRISMA) guidelines, MEDLINE, the China Journal Net, Web of science and Pubmed were searched from January 1990 to September 2021 for relevant articles. The following words used were ‘Papillary Thyroid Carcinoma’, ‘Ipsilateral’, ‘bilateral’ and ‘central neck lymph node dissection’. The list of references for the retrieved studies were reviewed to find any missing studies.

### Inclusion and exclusion criteria

The eligibility criteria for studies included in this meta-analysis were: 1) Prospective or retrospective studies. 2) These studies were published in English and Chinese. 3) Absence of lymph node metastasis based on preoperative imaging or intraoperative examination. 4) Available data about transient RLN injury, permanent RLN injury, transient hypoparathyroidism, permanent hypoparathyroidism and local recurrence.

The studies were excluded: 1) Studies were letters, case reports, reviews, and animal or laboratory research; 2) The studies with no control data were ruled out; 3) Duplicate studies were conducted on the basis of the same database. 4) Therapeutic CND or CND plus the lateral neck dissection.

### Data extraction

Both review authors independently chose inclusive studies and extracted data. The determination for inclusion in the analysis was taken by consensus. The full text copies of underlying associated studies were acquired. The next variables were documented: authors, gender, patients’ age, patients’ number and clinicopathological features.

### Statistical analysis

A formally designed meta-analysis of these included studies was performed to compare the outcomes of bilateral CND and ipsilateral CND for PTC. Our findings were transient RLN injury, permanent RLN injury, transient hypoparathyroidism, permanent hypoparathyroidism, and total recurrence.

The statistical analyses were undertaken using Review Manager 5.0. A fixed effects model was utilized to calculate pooled estimates of comorbidity, but a random effects model was applied based on heterogeneity. Homogeneity of effects was tested using the χ^2^ test, with *p* ≤ 0.05 indicating significant heterogeneity. When the homogeneity assumption was not rejected, a fixed-effects model was applied to assess the pooled effect of the outcome; when the opposite was true, a random-effects model was also carried out.

## Results

### Study selection

**Figure 1:**
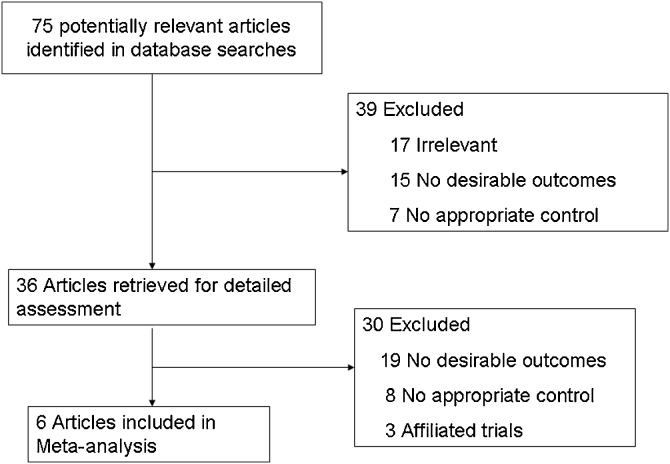
Flowchart of literature search results.

The flowchart of literature filtering with justification was showed in Fig. 1. 75 publications were available in the original search. 36 full-text research studies were evaluated for eligibility by excluding duplicates, irrelevant topics, non-original studies and studies without control groups. Finally, 6 studies^6^, ^7^, ^8^, ^9^, ^10^, ^11^ comparing ipsilateral CND with bilateral CND were eligible and included in our meta-analysis.

The basic characteristics of 6 included studies were shown in Table 1. All the studies were published from 2009 to 2020. Three studies in the Italy, 2 studies in Korea, and 1 study in USA. The study quality of 6 retrospective controlled studies was judged according to the NOS,^12^ with a scale distribution (0‒9 stars) of between 6 to 8 stars. All the enrolled studies revealed a comparatively high level of quality.

**Table 1:** Basic characteristics of included studies.

Author, year	Country	No. of patients	Sex (male/female)	Mean age (year)	Mean tumor size (mm)	Multifocality	Extrathyroidal extension	Time to recurrence (months)	Follow-up time (months)	Quality of score	Study design
Calò 2017^9^	Italy	258	56/202	44.4 ± 11.8	Ipsi-CND 16.7 ± 10.7	Ipsi-CND 18/30	‒	‒	Ipsi-CND 74.9 ± 19.2	7/9	Retrospective
					Bil-CND 13.8 ± 7.5	Bil-CND 21/30			Bil-CND 67.8 ± 22.8		
Giordano 2017^6^	Italy	610	135/475	49 ± 14	‒	‒	‒	‒	113 ± 53	7/9	Retrospective
Kang 2020^11^	Korea	174	0/174	Ipsi-CND 47.8 ± 11.2	Ipsi-CND 8 ± 5	Ipsi-CND 14/74	Ipsi-CND 43/74	‒	Ipsi-CND 118.2 ± 4.6	8/9	Retrospective
				Bil-CND 45.1 ± 10.2	Bil-CND 9 ± 6	Bil-CND 16/100	Bil-CND 66/100		Bil-CND 115.5 ± 4.1		
Raffaelli 2012^8^	Italy	186	37/149	42.9 ± 11.5	12.6 ± 6.8	93/186	‒	‒	25.1 ± 8.0	6/9	Retrospective
Sadowski 2009^10^	US	180	‒	‒	1.26	‒	‒	‒	38.8	6/9	Retrospective
Yoo 2018^7^	Korea	384	63/321	Ipsi-CND 48.25 ± 11.76	Ipsi-CND 10.38 ± 7.29	Ipsi-CND 22/169	Ipsi-CND 79/169	Ipsi-CND 29.33 ± 23.18	Ipsi-CND 58.91 ± 28.55	7/9	Retrospective
				Bil-CND 50.09 ± 12.56	Bil-CND 11.15 ± 6.48	Bil-CND 28/215	Bil-CND 113/215	Bil-CND 48.71 ± 17.46	Bil-CND 60.41 ± 28.67		

A total of 1792 patients underwent Ipsilateral CND or bilateral CNDforcN0 PTC were enrolled. RLN injury was evaluated by patient own self-assessment and postoperative laryngoscopy. Permanent RLN injury was considered as persistent hoarseness and vocal cord palsy identified by laryngeal examination more than 6 months postoperatively.^8^, ^11^, ^13^ Hypoparathyroidism was described as PTH levels < 10 pg/mL. Permanent hypoparathyroidism was determined as PTH < 10 pg/mL with constant low PTH levels throughout the follow-up period.^9^ Disease recurrence was ascertained clinically by ultrasonography and/or CT imaging, combined with serum Thyroglobulin (TG) levels.^6^

**Figure 2:**
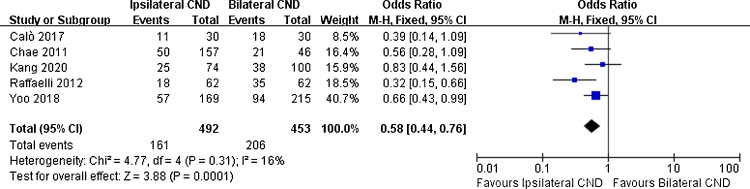
Fixed effects model for the Odds Ratios (ORs) and 95% Confidence Intervals (CIs) of transient hypoparathyroidism between ipsilateral CND group and bilateral CND group.

**Figure 3:**
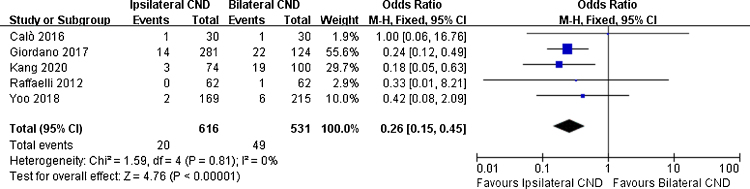
Fixed effects model for the Odds Ratios (ORs) and 95% Confidence Intervals (CIs) of permanent hypoparathyroidism between ipsilateral CND group and bilateral CND group.

There were 5 studies reporting transient hypoparathyroidism and permanent hypoparathyroidism. The incidence of transient hypoparathyroidism was significantly lower in the ipsilateral CND group than in the bilateral CND group (32.7% vs. 45.5% OR = 0.58, 95% CI [0.44∼0.76], *p* = 0.0001, Fig. 2). The occurrence of permanent hypoparathyroidism ipsilateral CND group was remarkably lower than that in bilateral CND group (3.2% vs. 9.2%, OR = 0.26, 95% CI [0.15∼0.45], *p* < 0.00001, Fig. 3).

**Figure 4:**
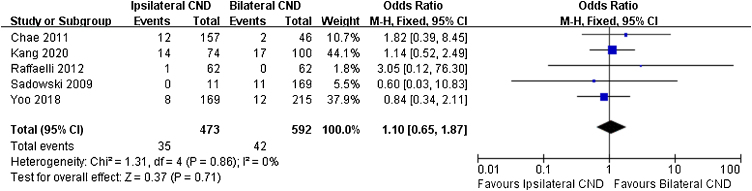
Fixed effects model for the Odds Ratios (ORs) and 95% confidence Intervals (CIs) of transient RLN injury between ipsilateral CND group and bilateral CND group.

**Figure 5:**
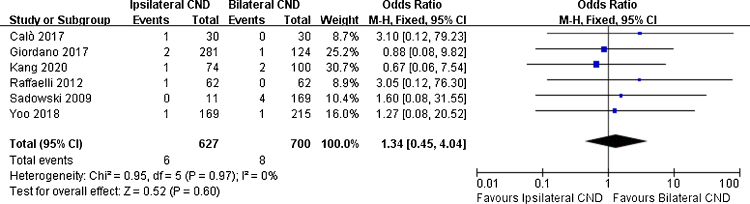
Fixed effects model for the Odds Ratios (ORs) and 95% Confidence Intervals (CIs) of permanent RLN injury between ipsilateral CND group and bilateral CND group.

Regarding transient RLN injury, there were 5 relevant studies. No significant difference in transient RLN injury was noted (7.4% vs. 7.1% OR = 1.1, 95% CI [0.65∼1.87], *p* = 0.71, Fig. 4). Permanent RLN injury was mentioned in 6 studies. No significant difference of permanent RLN injury was identified (0.9% vs. 1.1%, OR = 1.34, 95% CI [0.45∼4.04], *p* = 0.6, Fig. 5).

**Figure 6:**
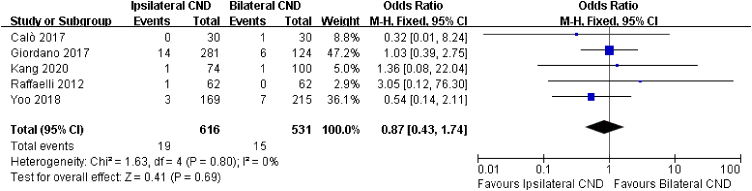
Fixed effects model for the Odds Ratios (ORs) and 95% Confidence Intervals (CIs) of local recurrence between ipsilateral CND group and bilateral CND group.

Local recurrence was described in 5 studies. No significant heterogeneity was found between the two groups (I^2^ = 0, *p* = 0.8), and there was no significant difference in local recurrence between the two groups (3.1% vs. 2.8%, OR = 0.87, 95% CI [0.43∼1.74], *p* = 0.69, Fig. 6).

## Discussion

Controversy remains over the surgical treatment of the central compartment of the neck in patients with cN0 PTC.^14^ As is widely known that unidentified, minimal or occult metastases may be discovered in 31%–62% of patients undergoing elective central neck dissection in the treatment of PTC, but the involvement of these lymph nodes seems to make little difference to clinical outcomes.^15^ In addition, one argument in favor of prophylactic CND is that it is difficult to define preoperative (through ultrasound and clinical examination) and intraoperative involvement of lymph nodes,^16^ despite the fact the surgeon’s evaluation of the central neck accurately predicts which PTC patients will receive benefit from CND.^17^ One of the chief debates against prophylactic CND is the higher risk of subsequent complications,^18^ even though supporters of prophylactic CND have illustrated that in the hands of experienced surgeons, prophylactic CND can be conducted without increased morbidity or complications.

For the purpose of reducing the potential risk of postoperative complications associated with prophylactic CND, ipsilateral CND has served as an acceptable alternative method to bilateral CND.^19^ Lee et al.^20^ reported that compared with bilateral CND, patients with ipsilateral CND had a lower frequency of transient hypocalcemia, 20% vs. 48% (*p* = 0.009), respectively. Our study suggested that the prevalence of transient and permanent hypoparathyroidism was significantly lower in the Ipsilateral CND group than bilateral CND group. During the procedure, the parathyroid gland may be affected by mechanical or thermal trauma, cut off blood supply, or inadvertent resection.^21^ Whereas there was no considerable difference in temporary/permanent RLN damage observed between these two groups. Our result was consistent with the previous study.^10^

Song et al.^22^ reported that the incidence of occult contralateral paratracheal node metastasis in the bilateral CND group was only 4.2%, also Lee et al. indicated that the total frequency of skipped metastases in their PTC group of patients was 6.8%.^23^ Given the infrequent metastasis of contralateral paratracheal nodes, ipsilateral CND was probably suitable for prophylactic CND forcN0 unilateral PTC. In our study, the local recurrence rate did not differ significantly between the Ipsilateral and bilateral CND groups. As a consequence, ipsilateral CND may be comparable to bilateral CND in terms of local recurrence.

According to these data, in cN0 unilateral PTC, we propose that ipsilateral CND may be safe and efficacious. Bilateral CND is not required because contralateral lymph node metastasis rate is below 20%^24^ and transient/permanent hypoparathyroidism is very high.

There are several limitations to our meta-analysis. Firstly, the current study is restricted by the shortage of well-quality RCTs and bias is unavoidable. Secondly, the choice to carry out a CND may be misguided by the surgeon’s preference. Thirdly, transient, and permanent hypoparathyroidism are not conventionally defined in our studies. Fourthly, the follow-up time of two studies^8^, ^10^ was less than five years, and only one study^7^ mentioned the time to recurrence. For these reasons, our research findings must be carefully interpreted.

## Conclusion

The permanent hypoparathyroidism in prophylactic ipsilateral CND was shown to be lower in our study compared to patients with bilateral CND. In addition, the local recurrence rate was not statistically different between the two groups. Our suggestion is that the outcomes of prophylactic ipsilateral CND in selected patients are better than the bilateral procedure.

## Funding

The present study was supported by HwaMei Research Foundation of Ningbo No.2 Hospital. Grant Number: No. 2022HMKY43.

## Conflicts of interest

The authors declare no conflicts of interest.
